# Celebrating the 150th anniversary of the *Descent of Man*

**DOI:** 10.1017/ehs.2021.15

**Published:** 2021-03-01

**Authors:** Sergey Gavrilets, Peter J Richerson, Frans B. M. de Waal

**Affiliations:** 1 Department of Ecology and Evolutionary Biology, Department of Mathematics, National Institute for Mathematical and Biological Synthesis, Center for the Dynamics of Social Complexity, University of Tennessee, Knoxville, TN 37996, USA; 2 Department of Environmental Science and Policy, University of California, Davis, CA 95616, USA; 3 Living Links, Yerkes National Primate Research Center, Emory University, Atlanta, GA 30322, USA

## Abstract

**Figure:**
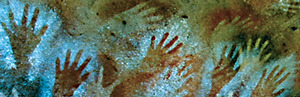

It has often and confidently been asserted, that man's origin can never be known: but ignorance more frequently begets confidence than does knowledge: it is those who know little, and not those who know much, who so positively assert that this or that problem will never be solved by science. (Darwin, *The Descent of Man*, 1871, p. 3).

The difference in mind between man and the higher animals, great as it is, certainly is one of degree and not of kind. (p. 85)

The formation of different languages and of distinct species, and the proofs that both have been developed through a gradual process, are curiously parallel … (p. 90)

A tribe including many members who, from possessing in a high degree the spirit of patriotism, fidelity, obedience, courage, and sympathy, were always ready to aid one another, and to sacrifice themselves for the common good, would be victorious over most other tribes … [A]s morality is one important element in their success, the standard of morality and the number of well-endowed men will thus everywhere tend to rise and increase. (p. 148)

This year we are celebrating the 150th anniversary of Darwin's book *The Descent of Man*, in which he laid down the foundation for scientific studies of human origins.

We now have a much better understanding of how new species arise and adapt to their environments – the process which Charles Darwin called a ‘mystery of mysteries’ (Darwin, 1859; Coyne & Orr, 2004; Gavrilets, 2004) – and we have begun to offer evolutionary accounts of the origin of our own species. Studying human origins necessarily implies a comparison with our close relatives, the other primates, and with other social animals. Research on human origins and our subsequent cultural and social evolution is vital not only for foundational scientific discovery but also for understanding, mitigating and solving *the most pressing challenges faced by our society*. Advancing research on human origins and social complexity is also very timely given *recent advances* and *emerging opportunities*. Fully capitalising on these requires a concerted *transdisciplinary effort* of researchers from a diverse set of disciplines. Success in this endeavor could yield tremendous gains for society.

## Challenges faced by our society

Understanding modern human behaviour, psychology, culture and many economic, political, and social processes is hardly possible without also considering the forces that have shaped our evolution (Reich 2018). For example, humans strongly react to inequality and injustice, a behaviour that we share with other primates (Brosnan & de Waal, 2014). Understanding the evolution of our sense of fairness may help us build a more just society. Similarly, studies of sexual selection (Anderson, 1994; Dixson, 2009) as well as genetic (Reich, 2018) and cultural (Mace & Jordan, 2011) diversity can shed light on gender, race and ethnicity-related prejudices, their consequences for human behaviour and ways to mitigate undesirable effects.

The genetic, physiological and psychological ‘machinery’ for our decision-making has evolved over time, shaped by forces like benefit and cost considerations (McElreath et al., 2008), social instincts (Richerson & Henrich, 2012), cognitive biases (Tversky & Kahneman, 1974; Haselton et al., 2005), beliefs (Gómez et al., 2017), normative values (Cialdini et al., 1990; Biccieri, 2006; Chudek et al., 2013), reinforcement (Baum, 2017) and the ‘theory of mind’ (Premack & Woodruff, 1978; Krupenye et al., 2016). Insights into the factors shaping human (pro)sociality can be leveraged to improve the efficiency and benevolence of collective actions in businesses and communities (Hofstede et al., 2010). They can foster more efficient economic, political, social and educational policies. Such insights can also be applied to better understanding of motivations leading to the onset and maintenance of (non)violent conflicts, which can increase societal resilience to external and internal shocks.

Evolutionary processes have also shaped human psychology influencing various aspects of our social life. Examples include religion and rituals, arts, sports and entertainment, activism and citizenship, leadership and followership, and the acceptance of or resistance to various innovations – cultural, economic, technological and political. Heavy reliance on cultural learning throughout our evolutionary history explains conformity (Song et al., 2012) and overimitation (Hoehl et al., 2019), as well as vulnerability to fake news and propaganda (Bernays, 1928; Atran, 2010). Understanding and managing societal changes will hardly be possible without taking all of these aspects into consideration (Henrich, 2016).

Our long-term persistence requires existential risks from climate change, biodiversity loss, depletion of non-renewable resources and security threats posed by unstable political systems to be addressed. Humans have evolved various psychological mechanisms and biases for making collective decisions, which need to be considered when developing policies for sustainability (Brooks et al., 2018). There are also various public health issues, including emerging epidemic diseases, addictions, obesity and geriatric disabilities, which are already benefiting from application of the emerging discipline of evolutionary medicine (Stearns & Medzhitov, 2015).

Artificial intelligence is rapidly becoming a pervasive fact of all of our lives. It brings not only technological advances but also threats that are difficult to predict. Research on the human brain and decision-making can be crucial in improving this technology. Simultaneously, improving human–artificial intelligence interactions requires a new look at human social psychology and on how we deal with various social dilemmas (Rahwan et al., 2019).

### Recent scientific advances

There has been an exciting cascade of recent discoveries related to human origins and evolution. We illustrate a large arc of progress with examples from primatology, paleoclimatology, brain evolution, genetics and social and cultural evolution. New transcriptome sequencing methods are allowing us to study the molecular and cellular differences in brain organisation between human and non-human primates (Sousa et al., 2017). Analysis of dynamic patterns of coordinated and anti-coordinated functional magnetic resonance imaging signals moves us closer to understanding the mechanistic correlates of consciousness (Demertzi et al., 2018). The capacity for cumulative culture has been crucial for the success of our species (Henrich, 2016), but cultural transmission of habits and knowledge is clearly not limited to humans as it is now being studied in animals including flies, fish, birds, primates and whales (Whitehead et al., 2019). The concept of ‘theory of mind’ comes out of ape studies in the 1970s (Premack & Woodruff, 1978), and after a period of doubt whether apes actually possess this capacity, it now seems that they understand that others’ actions are driven not by reality but by beliefs about reality, even if those beliefs are false (Krupenye et al., 2016). Palaeoclimatological and palaeoecological investigations are giving us an increasingly high-resolution understanding of the Pleistocene environments in which recent human evolution took place (Rodrigez et al., 2017). Genomic and archaeological studies are shedding further light on more recent events in human evolution, such as hybridisation between our ancestors and Neanderthals and Denisovans, the spread of agriculture and pottery in prehistoric Europe and population replacement in remote Oceania (Reich, 2018). These studies show a complex interplay of population migrations and the diffusion of cultural ideas across geographic landscapes. Archaeological and historical investigations increasingly produce sufficiently detailed time series that can be used to test sophisticated models of cultural evolution (Turchin et al., 2013; Currie et al., 2020). Experimental economics points to the crucial importance of social norms for human cooperative behaviour (Fehr & Schurtenberger, 2018); compliance with norms is conditional on the fact that others comply and are willing to punish free-riders (Gavrilets & Richerson, 2017; Gavrilets, 2020). Neuroeconomic studies of lesion patients with damage to different brain regions is allowing insights into the relative roles of intuition and deliberation in human cooperative behaviour (Wills et al., 2018). Cumulatively these and other advances give us a feeling that different pieces of a huge puzzle are starting to come together.

## Opportunities for further advances

Capitalising on recent advances can further the development of a unified theory of human origins and evolution. This can be more readily achieved by leveraging existing and growing public interest. Arguably, no area of life sciences is more compelling to the general public, as understanding of human origins and evolution underpins discussions of our place in the universe, of cognition and morality, and of our fate as a species.

## Going forward

Inquiry into human origins and evolution is among the greatest scientific challenges we face. Disciplinary pursuits can achieve singular discoveries, but more must be done. To move beyond the proverbial blind mens’ description of an elephant, we must blaze a new pathway forward through transdisciplinary collaborations of environmental scientists, biologists, social scientists and theoreticians. We must build on emerging trends in trans-disciplinary research to achieve truly transformative advances that can engross researchers, funders, legislators and the general public alike. Progress is being made to achieve this goal in evolutionary biology, where methods and insights from biology, mathematics, physics, chemistry and computational science have already become tightly intertwined. Social sciences are moving in a similar direction. This trend is especially visible in the establishment of several ‘synthesis centres’ by the National Science Foundation (NSF) in the last couple of decades. Each centre has been a tremendous success (Baron et al., 2017). Besides advancing research, these centres have nurtured a cadre of early career life and social scientists trained in transdisciplinary research. Making further progress will require further investment. With opportunity in sight and tools in hand, strategic injections of support from funding agencies like the NSF will be necessary to build momentum towards deriving a unified theory of human origins and social and cultural evolution.

In 1871 Darwin made a good start on a synthetic theory of human biological and cultural evolution. However in the late nineteenth and early twentieth centuries, many of his most notable synthetic ideas were lost as the social and life sciences became separate disciplines. With Darwin's work serving as a touchstone, the emerging synthesis pictures biology and culture as intricately entwined. Thus, what was old is once again new – an understanding worth celebrating for its own sake and for what it may inspire.

Darwin's book also introduced the concept of sexual selection, which continues to generate scientific insights of great interest and excitement to the general public including many that relate to dating, parenting, same-sex behaviour, arts and intelligence (Miller, 2000).

This Special Collection brings together eight papers on various aspects of human origins and cultural evolution. (We are extremely grateful to the authors who managed to prepare their manuscripts in spite of the challenges brought by the pandemic.)

The traditional concept of long and gradual, glacial–interglacial climate changes during the Quaternary has been challenged since the 1980s. High-temporal-resolution analysis of marine, terrestrial and ice geological archives has identified rapid, millennial- to centennial-scale, and large-amplitude climatic cycles throughout the last million years. These changes were global but have had contrasting regional impacts on the terrestrial and marine ecosystems, with in some cases strong changes in the high latitudes of both hemispheres but muted changes elsewhere. Such a regionalisation has produced environmental barriers and corridors that have probably triggered niche contractions/expansions of hominin populations living in Eurasia and Africa. Sanchez Goni (2020) review the long- and short-timescale ecosystem changes that have punctuated the last million years, paying particular attention to the environments of the last 650,000 years, which have witnessed key events in the evolution of our lineage in Africa and Eurasia. This review highlights, for the first time, a contemporaneity between the split between Denisovan and Neanderthals, at c. 650–400 ka, and the strong Eurasian ice-sheet expansion down to the Black Sea. This ice expansion could have formed an ice barrier between Europe and Asia that may have triggered the genetic drift between these two populations.

What were the selective forces responsible for humans becoming such a large-brained species relying on cooperation and social learning and how did it happen? A number of trigger hypotheses have been proposed which focus on a particular breakthrough innovation after which a positive feedback process drove relentless progress. These include bipedal hypothesis (Washburn, 1960), ecological dominance (Alexander, 1990), cooperative breeding (Hrdy, 2009), cooking (Wrangham, 2009), language (Maynard Smith & Szathmáry, 1995) and sexual selection for large brains (Miller, 2000). Wrangham (2021) discusses one such scenario that focuses on the idea that our species went through the process of self-domestication over the last 300,000 years by targeted conspiratorial killing of aggressive and dominant individuals.

The first studies of our closest ape relatives in the wild started in the 1960s in Tanzania by both Western and Japanese teams of scientists. These studies have provided a wealth of information relevant to questions of human evolution. A review of six decades of studies at Gombe National Park, started by British primatologist Jane Goodall and continued by many of her students, allows Wilson (2021) to speculate about the behaviour of the last common ancestor between the genera *Homo* and *Pan*, which lived 6–8 million years ago. In this comparison, another close relative, the bonobo, is equally relevant and complicates the picture because of its quite different behaviour (de Waal, 1997). For example, chimpanzees are male-dominated, whereas bonobo females are collectively dominant over the males. Wilson sees many parallels between chimpanzee behaviour (including their aggressive territoriality, tool-use, cultural abilities and slow development) and the behaviour assumed to have been present in our early ancestors. He plots the ways in which behaviour in the human lineage must have changed since then, including our bipedal gait, sexual division of labour, accumulative culture and extensive food transfers.

A study of young human children by Bailmel et al. (2021) seeks to answer the question of whether they seek to outmanoeuvre their partners or learn from them. Given the opportunity to do both or either, which outcome do they prioritise? Adam Baimel and co-authors subject children to multiple experiments and find that copying of observed behaviour has priority over the use of information for strategic purposes. The study also explores the cognitive capacities involved and finds that advanced perspective-taking abilities of children allow them to be better cultural learners, whereas these abilities have little effect on their chances to win. This is seen as evidence that in our evolutionary past the passing on of culture and information was probably more important than the use of social intelligence in a competitive context, even though the study cannot rule out that both played an important role.

Vilela et al. (2021) use ecological niche modelling to understand the evolution of agricultural subsistence systems on a global scale. Agriculture today is practised today in areas climatically and biotically similar to the original centres of plant and animal domestication in the early Holocene. Patterns are consistent with the hypothesis that the cultural descendants of early farmers have spread from the centres to the ecological limits of agricultural production in their continental or subcontinental regions. The limits of agriculture were for the most part reached millennia ago. At the frontiers agriculture becomes of secondary importance or is occasionally abandoned. Modern industrial technology has mostly improved agriculture within its historical limits rather than extending these frontiers. Only a handful of large areas (Western North America, Australia) became important agricultural producers only in the modern era.

Cultural evolution, like genetic evolution, occurs in the quotidian events of people or other organisms living out their lives, surviving changes (or not), passing on their genes and culture (or not). Wiessner and Pupu (2021) describe how the Enga of Highland New Guinea responded to the shock of modern innovations imposed on the Enga marriage institutions. Marriage institutions prescribe how mates and other kin mobilise resources to support women and their children. Children are the vehicles by which human genes and culture are transmitted to the next generation, making the evolution of marriage customs a critical part of the whole evolutionary process. The authors document in great detail how modern innovations such as cell phones and cheap transport undermined the complex arranged marriage system of the Enga. Young couples use modern technology to evade the traditional system, pursuing what they perceived to be their own interests at the expense of the wider social support system for child rearing. The ensuing marital breakdowns were managed by a new locally managed court system through which responsible adults in the community instituted reasonably effective new methods to engender responsible reproductive behaviour on the part of young couples.

Biological and cultural evolution have many features and processes in common. The ongoing loss of biological diversity as a result of anthropogenic factors is of great concern to scientists and the general public alike. Cultural diversity is also disappearing quickly. Zhang and Mace (2021) propose a theoretical framework to examine the phenomenon of cultural extinction which can be difficult to define and examine systematically. They show that over large millennial scales, cultural extinction can be studied in a phylogenetic comparative framework. Over decades or centuries, cultural extinction can be studied in a behavioural ecology framework. The authors review recent evolutionary studies that have informed cultural extinction processes and discuss fruitful directions for future research.

Starting with Plato, Confucius, Herodotus, Thucydides and Aristotle, political philosophers, historians, sociologists, economists and essayists have discussed and studied how human behaviour is shaped by interactions within society. These discussions have focused on five main questions: (1) what motivates human beings; (2) what constraints our natural and social environment imposes upon us; (3) what kind of society emerges as a result; (4) what constitutes a fulfilling life; and (5) what collective solutions can improve the outcome? Seabright et al. (2021) take a critical appraisal of the views held by some early influential social contract theorists (Hobbes, Locke and Rousseau) in light of recent cross-cultural empirical research of small-scale societies in the evolutionary social sciences. Seabright et al. (2021) conclude that social contract theorists severely underestimated human behavioural complexity in societies lacking formal institutions.

We hope that the papers in this Special Collection will stimulate further advances in the studies of human origins and cultural evolution.

## Data Availability

There is no data in this paper.
